# Exploring the Bioactive Mycocompounds (Fungal Compounds) of Selected Medicinal Mushrooms and Their Potentials against HPV Infection and Associated Cancer in Humans

**DOI:** 10.3390/life13010244

**Published:** 2023-01-16

**Authors:** Tomas Rokos, Terezia Pribulova, Erik Kozubik, Kamil Biringer, Veronika Holubekova, Erik Kudela

**Affiliations:** 1 Department of Gynecology and Obstetrics, Jessenius Faculty of Medicine, Comenius University in Bratislava, Mala Hora 4A, 036 01 Martin, Slovakia; 2 Department of Molecular Oncology and Diagnostics, Biomedical Center Martin, Jessenius Faculty of Medicine, Comenius University in Bratislava, Mala Hora 4C, 036 01 Martin, Slovakia

**Keywords:** HPV, cervical dysplasia, medicinal mushrooms

## Abstract

Medicinal mushrooms have been used as a medicinal tool for many centuries and, nowadays, are used in the prevention and therapy of various diseases, including as an adjunct to cancer treatment. It is estimated that 14–16% of global cancer cases are caused by infectious events; one well-known infectious agent that leads to cancer is the human papillomavirus (HPV). HPV is responsible for more than 99.7% of cervical cancer cases and also may play a role in vaginal, vulvar, penile, anal, rectal, and oropharyngeal carcinogenesis. *Coriolus versicolor*, a basidiomycetes class mushroom, consists of glycoproteins called polysaccharide-K (PSK) and polysaccharopeptide (PSP), which are mainly responsible for its effectiveness in the fight against a variety of cancers. Its beneficial effect lies in its ability to arrest different phases of the cell cycle, immunomodulation or induction of apoptosis. *Coriolus versicolor* extractcan reduces BCL-2 expression or increases the expression of p53 tumour suppressor genes in breast tumour cell lines. Inhibition of proliferation was also demonstrated with HeLa cells, while cervical cytology abnormalities improved in patients who locally applied *Coriolus versicolor*-based vaginal gel. *Coriolus versicolor* extract itself, and also its combination with another medicinal mushroom, *Ganoderma lucidum,* leads to improved HPV clearance in HPV cervical or oral-positive patients. Medicinal mushrooms can also increase the effectiveness of vaccination. This review considers the use of medicinal mushrooms as a suitable adjunct to the treatment of many cancers or precanceroses, including those caused by the HPV virus.

## 1. Introduction

It is estimated that 14–16% of global cancer cases are caused by infectious events while persisting virus infections are responsible for many of them [1], for example, the hepatitis B and C viruses in hepatocellular carcinoma [2]; the Epstein-Barr virus in Burkitt’s lymphoma [3]; and human papillomavirus (HPV) in cervical, vaginal, vulvar, penile, anal, rectal, and oropharyngeal cancers [4]. Cervical cancer is the fourth leading cause of cancer death in women worldwide [5], and more than 99.7% cases are caused by HPV [6]. HPV types are classified into four groups according to their carcinogenic potential, with 12 high-risk HPV (hrHPV) types [7]. After hrHPV DNA is incorporated into the DNA of the infected cell, oncogenic HPV proteins E6 and E7 are synthesized. These oncoproteins cause dysfunction of tumour suppressor proteins, leading to the dysregulation of the cell cycle, with neoplastic transformation of the affected tissue [8]. HPV types 16 and 18 cause over 70% of all cervical cancer cases worldwide. The most frequently detected oncogenic type is HPV16, followed by HPV18, HPV31, HPV52, and HPV58. In general, the highest incidence of HPV infection is in younger women, with the peak incidence occurring below the age of 25; incidence decreases with an increase in age. Such a decrease is not observed in developing countries [9].

## 2. HPV Infection in Humans

Papillomaviruses are small, circular, double-stranded DNA viruses. Persistent infection with oncogenic types of papillomaviruses can lead to the development of precancerous lesions and, later, to the development of cancer. The Papillomaviridae family contains 39 genera, and HPV can be found in five of them: *alphapapillomavirus*, *betapapillomavirus*, *gammapapillomavirus*, *mupapillomavirus*, and *nupapillomavirus*. The International Agency for Research on Cancer (IARC) classified HPV into groups according to carcinogenic potential: group 1 is carcinogenic for humans, group 2A is probably carcinogenic for humans, and group 2B is possibly carcinogenic for humans [10]. Thirteen HPV types belonging to groups 1 and 2A are responsible for up to 96% of cervical cancer cases [11]. Group 3 includes low-risk HPV types. Of the more than 200 known types, HPV groups 1 and 2A with oncogenic potential belong to the *alphapapillomavirus* genus, while HPV infection from the gamma and beta genera cause skin papillomas [12]. HPV can cause non-genital (cutaneous), mucosal or anogenital infections, or epidermodysplasia verruciformis. HPV infection can lead to laryngeal, oral, lung, and anogenital cancers [13].

Worldwide, HPV is the second most common cancer-causing infectious agent after *Helicobacter pylori*. About 5% of cancers are associated with high-risk HPV. During their lifetimes, 80% of the population will encounter HPV infection, but the majority of those will clear the infection without clinical symptoms. On the other hand, nearly all cervical cancer cases are associated with HPV infection. The prevalence of HPV infection in tumour tissues is estimated at 90% in the case of cervical and anal cancer, 70% in the case of vulvar and vaginal malignancies, and more than 60% in penile cancer cases. Oropharyngeal cancers are associated with tobacco and alcohol use, but 70% of them may be linked to HPV [14]. Table 1 provides these details.

## 3. Types of Medicinal Mushrooms and Their Biopotentials

For many years, mushrooms have been used as an effective therapeutic tool in the treatment of various diseases. For example, around 5300 years ago, Ice Man used amadou mushrooms (*Fomes fomentarius* (L.) Fr.) to survive in the inhospitable conditions of the Italian Alps. Hippocrates also described this mushroom as a potent anti-inflammatory treatment. On the other side of the world, the first inhabitants of North America used puffball mushrooms (*Calvatia* genus) to improve the wound healing process [16]. The people of Asia have also used mushrooms as a medicinal tool for many centuries. Nowadays, medicinal mushrooms have been approved in eastern countries as an adjunct to cancer treatment. Commonly used species include *Ganoderma lucidum* (Curtis) P. Karst, *Lentinus edodes* (Berk.) Singer, and *Trametes versicolor* (L.) Lloyd, which is also called *Coriolus versicolor* or turkey tail. Medical mushrooms are also distributed in other parts of the world, but in the US, for example, they are distributed as dietary supplements and regulated as food, not drugs. Manufacturing consistency is not controlled for dietary supplements, so it is not possible to guarantee that a product contains the ingredients listed on the label. The US Food and Drug Administration (FDA) has these dietary supplements as treatments for any medical condition [17].

Many countries fail to regulate the handling of medicinal mushrooms and their components, which can lead to a reduced content, a lack of effective components in the sold supplements, or even replacement of the effective components by others that can have an adverse effect on human health. Due to the fact that the fungal extract may contain a large spectrum of demonstrably or potentially bioactive compounds, it is difficult to monitor the effectiveness of sold supplements. Determining the exact dose of a substance whose beneficial effect on human health could be incorporated into a study is challenging. Therefore, it is difficult to prove the effectiveness of medicinal mushrooms; however, despite the lack of evidence, their beneficial effect on human health has been known for a long time [18].

Many bioactive compounds such as polysaccharides, proteins, fats, minerals, glycosides, alkaloids, volatile oils, terpenoids, tocopherols, phenolics, flavonoids, carotenoids, folates, lectins, enzymes, and ascorbic, and organic acids are found in medicinal mushrooms and are responsible for more than 100 medicinal functions. The most important of these functions are antioxidant, anticancer, antidiabetic, antiallergic, immunomodulating, anticholesterolemic, antiviral, antibacterial, antiparasitic, antifungal, detoxification, anti-inflammatory, and hepatoprotective effects [19]. Medicinal mushrooms are mainly used as dietary supplements or functional foods, but they have potential as drugs for traditional and/or evidence-based medicine. The most important mushroom species mentioned in research are *G. lucidum*, *C. versicolor*, *Lentinula edodes* (Berk.) Pegler, *Agaricus brasiliensis* (Wasser et al.), *Cordyceps sinensis* (Berk.) Sacc., *Grifola frondosa* (Dicks.) Gray, *Hericium erinaceus* (Bull.: Fr.) Pers., and others [20].

## 4. Mechanism of Cell Proliferation and Immunomodulation Properties

The effectiveness of *C. versicolor* polysaccharides is well documented. Several studies have demonstrated the effectiveness of *C. versicolor* in the fight against a variety of cancers, mostly using polysaccharopeptide (PSP) and polysaccharide K (PSK) called krestin, extracted from this mushroom. They have proven to be helpful in ovarian [21], cervical [22], prostate [23], colon [24], lung [22], and breast [25] cancer treatment, as well as in the fight against leukemia [26] and other cancers.

The protein extract of this mushroom can cause cell cycle arrest [27]. It can also affect apoptotic pathways. Proteins BCL-2 and BCL-X_L_ are BCL-2 family proteins, which are regulators of the mitochondria-mediated apoptotic pathway. While BH3-only proteins, BAK, and BAX are pro-apoptotic, BCL-2 and BCL-X_L_ have anti-apoptotic function [28]. In breast cancer cells, 17β-estradiol stimulates overexpression of BCL-2, which decreases levels of mitochondrial apoptotic factors [29]. *C. versicolor* extract demonstrably reduces BCL-2 expression in breast cancer cells. An increased expression of genes for tumour suppressor protein p53 has also been observed in some breast tumour cell lines incubated with *C. versicolor* extract [30]. The cytotoxic effect of *C. versicolor* protein-bound polysaccharides on melanoma cells has also been confirmed via increased intracellular reactive oxygen species [31].

Caspase-3 is a death protease, one of the crucial mediators of apoptosis. Its precursor, precaspase-3, has at least 200-fold less activity than caspase-3. The overexpression of this precursor was confirmed in cancer tissue [32]. The genes of this precursor are the target of the E2F family of transcription factors. E2Fs are in an inactive form due to binding with the retinoblastoma protein (Rb) [33]. The dissociation of this bond leads to the excessive activity of the transcription factor. The dysregulation of the cell cycle based on this dissociation has been demonstrated in multiple cancers while the oncogenic potential of the E7 HPV protein also lies in this mechanism. This pRb/E2F pathway dysregulation leads to the eventual upregulation of gene transcription for precaspase-3 [32]. In promyelomonocytic leukemia cells, PSK activates caspase-3, which leads to the induction of apoptosis [34]. In the field of neurotoxicity, *C. versicolor* aqueous extract was found to have protective value in nitric oxide-induced brain diseases due to its effect on caspase-3 enzyme activity [35].

The Nuclear Factor kappaB (NF-κB) is in the transcription factor family; these affect immune response and inflammation and determine expression of p53 tumour suppressor protein genes or genes for signal transducers and activators of transcription (STAT) [36].

In interferon (IFN) signaling, after binding pathogen-associated molecular patterns (PAMP) to pathogen recognition receptors (PRR), interferon-regulatory factors drive expression of IFN genes [37]. In the next step, IFN binds to its receptors, leading to STAT activation. IFN molecules bind to cell surface receptors and initiate a signaling cascade through the Janus kinase signal transducer and activator of transcription (JAK-STAT) pathway, leading to the transcriptional regulation of hundreds of IFN-regulated genes [38]. STAT promotes expression of interferon stimulated genes (ISGs), which mediate antiviral responses [39]. STAT1-regulated genes are important targets of host gene regulation by HPV [40]. For example, HPV31 E7 can suppress STAT1 at the transcriptional level, resulting in reduced IFN-mediated gene expression [41]. HPV16 E7 inhibits IFN-induced phosphorylation, the nuclear translocation of STAT1, and the downstream expression of ISGs [42]. It has also been established that overexpressed E6 and E7 in keratinocytes repress the expression of innate immune genes [43].

Ethanolic extract of *C. versicolor* reduces prostate cancer cell growth. An in vitro study showed that this extract increased the levels of STAT1, a possible mechanism of its action [44]. On the other hand, *C. versicolor* extract showed anti-inflammatory effects in mice model inflammatory bowel disease by reducing STAT1 and STAT6 expression, leading to lower IFN-γ and interleukin-4 (IL-4) expression [45].

The immunostimulatory effects of PSP were demonstrated in animal models, through elevation of pro-inflammatory cytokines like IL-6 and tumor necrosis factor α (TNF- α) [46]. PSP in simultaneous activation with antigens, such as lipopolysaccharide bacteria wall components, leads to activation of the PRR toll-like receptor 4 (TLR4),which increases IL-6 production. Induction of the TLR4 signalling pathway also leads to the activation of NF-κB [47]. These two inducers may also activate the signalling pathway via STAT3 [48]. On the other hand, incubation of human leukemia cells with aqueous extracts of *C. versicolor* leads toa decrease in transcription factor NF-κB and a decrease in the expression of cyclooxygenase 2 (COX-2), whose products are responsible for higher levels of cell proliferation and angiogenesis and the reduction of apoptosis. A study of *C. versicolor* extract on human leukemia cells also shows STAT1 elevation [49].

PRR ligands such as N-acetyl glucosamine, beta glucans, and lipopolysaccharide activate innate and adaptive immunity by binding to receptors such as TLR4 or complement receptor 3. This leads to the secretion of inflammatory cytokines like IL-6 or TNF-α [50]. PSK through TLR4 plays a role in the activation of TNF-α secretion [51]. Another work describes two possible routes of *C. versicolor* extract‘s effect on pro-inflammatory cytokine expression. Secretion of cytokines IL-6 and TNF-α by macrophages and TLR4 expression were stimulated by the extract itself. Additionally, during treatment of cells with lower concentrations of lipopolysaccharide, the extract increased cytokine production, while higher dose of lipopolysaccharide led to their reduced synthesis. In other words, *C. versicolor* extract showed an antagonistic or additive effect according to lipopolysaccharide concentration [52].

*Pleurotus ferulae* [53] is another medicinal mushroom, which affects immunological response. By improving maturation and function of dendritic cells, it helps to link innate and adaptive immunity. T and B lymphocytes with antigen-specific surface receptors play an important role in adaptive immunity. Lymphocyte effector clones are formed after antigen binding to lymphocyte receptors. Cytotoxic T-lymphocytes and NK cells are the main parts of innate immunity in the immune response against viral pathogens [54]. The major histocompatibility complex (MHC) plays an important role in the process of the activation of T and B lymphocytes. By MHC, class I processes endogenous antigens as viral proteins produced by the cell. They are marked in cytoplasm by ubiquitin and are destroyed by proteasomes. Subsequently, they are moved to the endoplasmic reticulum, where α chain and β2microglobulin are synthesized, then transported to the Golgi complex, and finally transported to the cell surface, where they are recognized by CD8+ T lymphocytes. After binding CD8+ to MHC, class I CD8+ form a receptor for IL-2 and, with the help of the Th1 subpopulation of CD4+ T lymphocytes, CD8+ lymphocytes mature into mature cytotoxic Tc lymphocytes. Tc lymphocytes release perforins and granzins from cytotoxic granules—enzymes that lead to apoptosis of the target cell. Thus, after recognizing tumor cells or cells attacked by intracellular microorganisms, especially viruses, Tc lymphocytes cause their degradation.

MHC class II molecules play role in the processing and presentation of external molecules that have entered the cell by endocytosis or phagocytosis. These are antigen presenting cells—dendritic cells, monocytes, macrophages, and B-lymphocytes. After processing antigens in the endolysosome fragments, they bind to the MHC II molecules. Such a complex is transported to the cell surface and is subsequently recognized by CD4+ T lymphocytes. Subsequently, Th lymphocyte precursors are produced, which further develop into the next subpopulations. If the precursors develop in the presence of cytokine IL-12, they differentiate into the Th1 subpopulation. The Th2 subpopulation arises in the presence of IL-4 and the Th17 subpopulation is formed in the presence of IL-1 or IL-6. Subsequently, formed Th1 cells mainly produce IFN-γ and IL-2, Th2 cells produce cytokines IL-4, IL-5, IL-10, IL-13, and also influence the maturation of B lymphocytes into plasma cells and memory B cells. Th17 cells produce IL-17 and influence the production of pro-inflammatory cytokines and chemokines. Protection against intracellular microorganisms is ensured by Th1 cells [54,55].

## 5. Mechanism of Anti-HPV Properties and Vaccination Support

Patients affected by pre-cancerous changes of the cervix can also benefit from the use of *C. versicolor* products. A retrospective observational study evaluated the efficacy of *C. versicolor*-based vaginal gel in 183 high-risk HPV-positive women with normal or abnormal cytology. The patients applied vaginal gel for three months and were HPV DNA tested after six months. HPV negativity was confirmed in 67% of patients who applied the gel versus 37.2% of the control group. Furthermore, cytology improvement was observed in 78.5% of the treated patients versus 37.7% of controls [56]. Another study enrolled 91 HPV-positive women with low-grade Pap smear lesions. Normal Pap smears performed three months after treatment were obtained in 78% of patients in the treated group, compared to 54.8% in the control group. At their six-month visits, the high-risk HPV group showed 62.5% HPV clearance in those who applied the gel versus 40% in the control group [57]. Both studies demonstrated higher cytology improvement and HPV clearance in patients who applied *C. versicolor*-based vaginal gel. The effect of *C. versicolor* on HPV clearance was also confirmed for oral HPV infection; 61 patients underwent oral swabs for gingivitis and were positive for HPV16 or HPV18. They took capsules containing Mycelia extract from medical mushrooms *Laetiporus sulphureus* (Bull.) Murrill and a combination of extracts from *T. versicolor* and *G. lucidum* for two months. HPV was cleared in 87.8% of patients who took *T. versicolor* and *G. lucidum* extract while it was cleared in only 5% of the patients treated with *L. sulphureus* [58].

The immune system is one of the basic systems important for maintaining the homeostasis of the organism and its defense against environmental factors. Organisms, molecules or parts of molecules represent antigens that the immune system recognizes and triggers an immune response. The immune response is stimulated after the interaction between the antigen and the receptor, while the innate immune mechanisms are the first involved in defense reactions [54]. Infectious agents release PAMPs, which are recognized by receptors on the surfaces of the epithelium, that lead to the activation of the cellular and humoral mechanisms of innate immunity [59]. To recognize PAMPs, the innate immunity uses PRR, which are coded in the genome, and no further modification is required for their use. The PRRs recognize the patterns found on pathogens while such patterns are not found on the body’s own cells, so innate immunity can distinguish its own structures from foreign ones. From a functional point of view, PRRs are divided into several groups while the best known are Toll-like receptors (TLRs). These are divided into 10 groups according to the ligands they can recognize [54]. Canella F. et al. [60] quantified TLR-2, 3, 4, 7, and 9 transcripts in HPV-positive and HPV-negative cervical samples from 154 women. Higher expression of TLR-9 was proved in HPV-positive samples, and extremely higher levels of this receptor were observed in patients with persistent HPV infection in this study [60]. On the other hand, oncoproteins E6 and E7 are able to block TLR-9 induced cytokine production in keratinocytes. The mechanism of this inhibition was demonstrated by in vitro infection of keratinocyte cells with HPV16 virions. After 24 h, the expressed oncoprotein E7 caused the formation of a nuclear complex consisting of estrogen receptor 1 (ESR1 also ERα) and a dimer of two members of the NF-κB family of transcription factors (NFKB1 and RELA or p50 and p65) under the influence of IκB kinase (IKK). This complex binds to the DNA region of the TLR-9 promoter, thereby preventing the initiation of gene expression for this protein. In addition, the NF-κB family member RELA (p65) together with ERα interaction with histone deacetylase 1 (Histone Deacetylase 1–HDAC1) and lysine specific demethylase (Lysine (K)-Specific Demethylase 5B–KDM5B also JARID1B) caused histone modification of the TLR-9 promoter. These processes caused the suppression of TLR-9 transcription with a subsequent impact on weakening the function of innate immunity, mainly by reducing the production of IFN1 [61].

*Macrocybe lobayensis* (R. Heim) Pegler & Lodge, from the Tricholomataceae family, has been used for centuries in traditional medicine as well. A heteroglycan protein with a strong antitumor and immunomodulatory effect was isolated from this mushroom [62]. Such extract rich on polysaccharides from this mushroom is able to upregulate the expression of TLR-2 and TLR-4 [63]. Canella at al. [60] did not demonstrate a higher expression of these two receptors in low-risk and high-risk HPV positive cervical cells collected with a cytobrush from both ectocervix and endocervix samples while a study by Daud I. et al. [64] showed in endocervical specimens 80-fold greater TLR-2 the median positive change in women who cleared HPV16 infection than women who persisted this infection [64]. Although the overexpression of specific TLRs in HPV infection is disputable, the immunomodulatory effect of the *M. lobayensis,* caused by augmented macrophage activity and the TLR signalled modulated expression of immunomodulation-related genes including NF-κB, COX-2, IFN-γ, TNF-α, and Iκ-βα, stimulates the immune system in a fight against pathogens causing the infection [63].

HPV vaccination is an effective method of primary prevention, but its sufficient effect on already developed HPV-associated cancer has not been confirmed. On the other hand, anticancer immunotherapies have presented great development in recent years. In gynecology cancer, the two main ways of immunotherapy are promising—monoclonal antibodies in function of immune check-point blockers and T cell-based immunotherapy [65]. Dendritic cells are used in antitumor vaccines, mainly due to their ability to activate naive CD4 and CD8 T cells [66]. The positive effect of *P. ferulae* polysaccharides on the antitumor therapeutic HPV dendritic cells-based vaccine was proved in an animal model. HPV dendritic cells-based vaccine supported by *P. ferulae* polysaccharides significantly inhibited tumor growth with the increased activation of CD4+ and CD8+ T cells. Polysaccharides of this mushroom improved the antitumor efficacy of therapeutic vaccine [67]. Roopngam et al. [68] proved higher amounts of T-lymphocytes in the group of T-lymphocytes cocultured with the dendritic cells pulsed by the HPV16-E7 proteins and treated with *Pleurotus sajor-caju*-β-glucan polysaccharides in comparison with T-lymphocytes without this treatment. This work suggests that *P. ferulae* polysaccharides is a suitable tool for the effective improvement of vaccines in cervical cancer [68]. Another work analysed *P. ferulae* water extract effect on the maturation and function of dendritic cells. Authors observed the induction of antigen-specific CD8+ T cell responses in HPV E6 and E7 peptides pulsed dendritic cells while cells treated with *P. ferulae* water extract showed higher level of CD8+ T cell responses and caused higher tumor growth inhibition [53].

Another mushroom used in traditional Chinese medicine, *Flammulina velutipes* (Curtis) Singer, showed immunomodulating effect in a mice model. Fungal protein isolated from this mushrrom stimulates maturation of dendritic cells and induce antigen-specific CD8+ T-cell immune responses. This study used the HPV16 E7 oncoprotein as an antigen and finally suggests *F. velutipes* fungal protein as a suitable adjuvant for cancer immunotherapy [69].

## 6. Mechanism of Anti-Cancer Properties

The in vitro study of *C. Versicolor* PSK’s anti-tumour activity evaluated its effect on various tumour cell lines, including human cervix adenocarcinoma (HeLa) cells. Tumour cell lines were cultured with PSK or in medium alone. Inhibition of proliferation was demonstrated in tumour cell lines. In the case of HeLa cells, the inhibition rate (57%), in correlation with the control, was higher at a lower concentration of PSK (50 μg/mL vs. 100 μg/mL). Cell cycle phase distribution analysis showed partial accumulation of HeLa cells in the G0/G1 phase and a decreased number of cells in the S phase and G2/M phase. In human gastric cancer cells, detectable active caspase-3 protease was present in 36% of PSK-treated cells; this effect was not found in HeLa cells [70]. Knežević et al. demonstrated the antitumour effect of *C. versicolor* on HeLa cells [71]. This work showed a stronger effect from mycelium extracts than basidiocarp extract on HeLa, human colon carcinoma, and human lung adenocarcinoma cell lines. The HeLa cells were the most sensitive to the extracts [71].

*G. lucidum* is a medicinal mushroom known as lingzhi in China and reishi in Japan. It has been used for many years in traditional Chinese medicine due to its many beneficial effects on human health [72]. Among other benefits, it has been used as an alternative adjuvant therapy for cancer [73]. *G. lucidum* consists of several components; polysaccharides and triterpenes are responsible for its antitumour effect [74,75]. Polysaccharides composed of α/β-glucans, glycoproteins, and water soluble heteropolysaccharides show antitumour effects by various mechanisms; these include immunomodulation and antioxidation, as well as anti-proliferative, pro-apoptotic, and anti-angiogenic functions [76,77]. *G. lucidum* extract also showed antitumour activity in cervical cancer cells, especially with the inhibition of proliferation and induction of apoptosis. Aqueous extracts from Chinese and Mexican *G. lucidum* samples were incubated with HeLA, SiHa, and C-33A cancer cells. Inhibition of proliferation was confirmed in all tested cell lines. SiHa cells treated with *G. lucidum* from Mexico showed the highest cytotoxic effect. An analysis of the effects of 320 µg/mL aqueous extract from this mushroom on the cell cycle showed cell cycle arrest at the G2/M phase in HeLa and C-33A cancer cells while SiHa cells arrested the cell cycle in the G0 phase. *G. lucidum* induced growth inhibition of cells transformed by HPV can be reached via apoptosis. HeLa, SiHa, and C-33A cells treated with this extract showed the formation of DNA laddering, so the antitumour effect of *G. lucidum* might also be caused by the induction of apoptosis [78].

In addition to polysaccharides, triterpenoids are also involved in the antitumour effect of *G. lucidum*. In one study, the separation of triterpenoid enriched extract was performed, and individual triterpenoids ganolucidic acid E, lucidumol, ganodermanontriol, 7-oxo-ganoderic acid Z, 15-hydroxy-ganoderic acid S, and ganoderic acid DM were obtained. The cytotoxic effects of these triterpenoids were tested on three tumour cell lines, including HeLa cells. All six isolated triterpenoids were able to reduce cell growth while 15-hydroxyl-ganoderic acid S exhibited the most cytotoxicity in HeLa cells. All six compound treatments showed sub-G1 accumulations in HeLa cells [79]. When in the process of apoptosis, the execution pathway is initiated by caspase-3 cleavage; the degradation of chromosomal DNA occurs while fragmented DNA multimers leak out of the cell. This results in a DNA content reduction in cells, which can be detected with special staining; these apoptotic cells are represented by a sub G0/G1 population [80]. This is how the induction of apoptosis by *G. lucidum* triterpenoids was observed in HeLa cells [79]. The tumour suppressor function of PSK in *C. versicolor* is similar to the effect of *G. lucidum* polysaccharides, as shown in cervical tumour-bearing mice. After treatment with enzymatically hydrolysed *G. lucidum* polysaccharide, they showed decreased expression of Bcl-2 and COX-2 and increased expression of Bax and cleaved caspase-3 [81].

Jin et al. [82] also demonstrated *G. lucidum* polysaccharide‘s antitumour effect on cervical cancer cells. A polysaccharide from this mushroom promoted the apoptosis of cervical cancer cells and attenuated their invasion and migration abilities. Western blot assay analysis of these cells showed a higher expression of pro-apoptotic proteins Bax and caspase-3 and a lower expression of anti-apoptotic protein Bcl-2 [82]. The phosphorylation of STAT5 protein increased with the severity of cervical intraepithelial neoplasia (CIN) while higher levels of phosphorylated STAT5 were observed in HPV16 and HPV18 positive cancer cells than in HPV-negative cancer cells [83]. HPV oncoprotein E6 induces the phosphorylation of the JAK2-activating STAT5 and STAT3. The increased severity of CIN also increases activation of both these proteins. The opposite relation is also described, where the silencing of STAT5 and STAT3 leads to the decrease in the viral oncoproteins E6 and E7 expression [84]. Jin et al. [82] proved the decreased expression of phosphorylated-JAK and phosphorylated-STAT5 in cervical cancer cells, which were treated with *G. Lucidum* polysaccharide [82].

Another medicinal mushroom, *Cordyceps sinensis* (Berk.) Sacc., has been used in Chinese traditional medicine for the prevention or treatment of many diseases, including cancer. One study described its beneficial effect in uterine cervical cancer in mice [85]. In this work, selenium enriched *C. sinensis* was used, as selenium administered to laboratory animals shows a protective effect against tumour formation [86]. The study showed significantly longer survival of animals receiving selenium enriched *C. sinensis* in comparison with animals receiving just selenium or *C. sinensis*. The shortest survival time was observed in the no treatment group [85].

Medicinal mushrooms can also improve oncological treatment, not only by their own effects but also by increasing the effects of radiotherapy or chemotherapy itself. *Pleurotus ostreatus* (Jacq.) P. Kumm. is widely used in the prevention of many diseases and in meat product correction as a novel ingredient [87]. Ergosterol peroxide isolated from *P. ostreatus* showed a loss of viability in HeLa and CaSki cervical cell lines with its increased dose. This work suggests that ergosterol peroxide isolated from *P. ostreatus* can serve as a radiosensitiser in cervical cancer treatment [88]. Lung cancer cells pretreated with another medicinal mushroom, *Lentinus squarrosulus* Mont., showed amplified cisplatin-induced apoptosis. Some downstream signals, which lead to changes in Bax, Blc-2, and p53 expression, showed higher levels of apoptosis in lung cancer cells preincubated with peptide from *L. squarrosulus.* This suggests use of this medicinal mushroom may be a suitable supplement to chemotherapy with cisplatin in lung cancer treatment [89].

## 7. Selected Medicinal Mushrooms and Bioactive Compounds

Polysaccharide-protein complex (PSPC) is a heteropolymer isolated from the culture filtrates of *M. lobayensis.* It is a protein-bound polysaccharide whose protein part is made up mainly of acidic amino acids, such as aspartic and glutamic acids [62]. In addition, *P. ferulae* water extract improves the maturation and cytokine production. This extract enhances the proliferation of CD8+ T-cells and antigen presentation through dendritic cells [53]. Moreover, the major fruiting body protein of *F. velutipes* is an acetylated protein consisting of 114 amino acid residues, which is similar to *G. lucidum* bioactive compounds [69]. In particular, *P. ferulae* ergosterol peroxide leads to loss of viability in HeLa and CaSki cervical cell lines according to its dose in vitro and may be effective as a radiosensitizer in treating cervical cancer [88]. Additionally, purified peptides from aqueous extracts of *L. squarrosulus* increase cisplatin-induced cytotoxicity in human lung cancer [89]. The genus *Cordyceps* consists of many compounds, such as proteins, cyclic peptides, polyamines, nucleosides, polysaccharides, and sterols, while the major bioactive compounds are nucleosides cordycepin and its analogues, polysaccharides, and sterols [90]. Table 2 provides an overview of these compounds.

Turkey tail PSK consists of mixture of glycoproteins whose main element is beta glucan and polysaccharopeptide (PSP) [91]. Other small molecular weight components like flavonoids are present in *C. versicolor* composition, but these are not the main parts. The principal monosaccharide of PSP and PSK is D-glucose while they contain individual variabilities in sugar compositions [98].

Mishra et al. [99] described a positive effect of *Pleurotus* spp. on breast, colorectal, cervical, and hepatocellular carcinoma cells. The spectrum of molecules within *Pleurotus* spp. Contain, such as α-glucans, β-glucans, lentinan, lipopolysaccharides, resveratrol, Cibacron Blue affinity purified protein, concanavalin A, and others, can affect various signalling cascades responsible for inhibition of growth, proliferation, angiogenesis, and metastasis in cancer cells [99]. Anticancer effects can be provided by cell cycle arrest in the pre-G0/G1 phase, higher production of nitric oxide by macrophages, and increased cytotoxicity of NK cells [100].

Another bioactive compound, C. versicolor PSP (Figure S1) [101] is mainly composed of β-glucans responsible for immunomodulation by its effect on cytokine release, enhanced dendritic and T-cell infiltration into tumours, overexpression of cytokines and chemokines, and NK cells activation. PSP also shows antitumor, anti-inflammatory, and antiviral effects [102]. C. versicolor proteoglycan PSK (Figure S2) [103] is also composed mainly of β-glucans with similar activities as PSP. Many articles are available describing the benefit of these two proteins, but the exact mechanisms of their function are not fully understood [104].

Additionally, the antiproliferative effect of *G. lucidum* extracts containing triterpenoids is well known; however, the detail function depends on the cell type and treatment method. The chemical structure of *G. lucidum* triterpenoids is an oxygenated lanostane. According to structure, they can be divided into roughly ten groups. Wu et al. [105] described seven anticancer effects of *G. lucidum* triterpenoids (Figure S3). These can affect the cell cycle, downregulate gene expression of proteins responsible for proliferation signaling, deactivate telomerase and DNA topoisomerases, inhibit inflammation, induce apoptosis and autophagy, suppress cell migration and invasion, and provide anti-angiogenetic activity [105].

*L. edodes* is another medicinal mushroom used extensively in eastern countries. Its β-1,3-D-glucan called lentinan (Figure S4) [106].plays a role in immunomodulation. By binding with pattern recognition receptors, it affects immunity response [107]. Lentinan also shows cytotoxic effects by inducting apoptosis through intracellular reactive oxygen species [108]. The bioactive compound of *C. sinensis* is called cordycepin (Figure S5), also known as 3-deoxyadenosine, and is responsible for its anticancer effect [95]. According to its structural similarity with adenosine monophosphate, where cordycepin lacks the 3′-hydroxyl group of the ribose moiety [109], it can be used by DNA and/or RNA polymerases [110] and cause the termination of nucleic acid elongation [111].

*L. squarrosulus* is another wild mushroom with anticancer activity with bioactive compounds: 1-tetradecene, 9-eicosene, phytol, octahydropyrrolo[1,2-a]pyrazine (Figure S6), fumaric acid, monochlorid, 6-ethyloct-3-yl ester (Figure S7), and 3-trifluoroacetoxypentadecane [112]. Benefits of this mushroom lie in changes in Bax, Blc-2, and p53 expression and higher levels of apoptosis in lung cancer cells preincubated with a peptide from *L. squarrosulus* [89].

Several studies point to the immunomodulating and antiproliferative effects of medicinal mushrooms, which suggest their use as adjuvant treatment for some cancer diseases and can also increase the effectiveness of vaccination. On the other hand, most of the published papers were performed in vitro or on animal models while in vivo studies are lacking. Many of the published studies also point out the effectiveness of polysaccharide mixtures obtained from mushroom extracts, but defining a specific pure molecule responsible for the beneficial effect of medicinal mushrooms is often difficult.

## 8. Conclusions

Medicinal mushrooms have been used for thousands of years in the traditional medicine of many countries due to their curative and preventive effects on various diseases. Today, a number of works describe the functional components of fungi in the fight against many diseases, including cancer. In the case of cervical cancer, the beneficial effects of medicinal mushrooms on hindering the development of the disease, mainly due to cell cycle arrest and induction of apoptosis, have been proven. In the case of cervical precancerous lesions, an increased HPV clearance and improved cervical cytology were demonstrated in patients applying vaginal gel during the watchful waiting period. Medicinal mushrooms appear to be a suitable adjunct to the treatment of many types of cancer, and patients with diagnosed precancers can also benefit from their use.

## Figures and Tables

**Table 1:** Percentage of HPV associated malignancies and HPV prevalence [14,15].

Affected Tissue	Percentage of HPV Associated Cancers in Women and Men	HPV Prevalence in Affected Tissue
Cervix uteri	49% in female HPV-associated cancers	90%
Vagina	3% in female HPV-associated cancers	70%
Vulva	16% in female HPV-associated cancers	70%
Penis	7% in female HPV-associated cancers	60%
Anus	18% in female HPV-associated cancers 12% in male HPV-associated cancers	90%
Oropharynx	14% in female HPV-associated cancers 81% in male HPV-associated cancers	70%

HPV: Human papillomavirus.

**Table 2:** Bioactive compounds, efficacy and edibility of selected medicinal mushrooms.

Source	Bioactive Compounds	Efficacy	Edible/Toxic
*T. versicolor* [91]	PSK, PSP	Cell-cycle arrest, affects apoptotic pathways, increases ROS	Edible [92]
*G. lucidum* [77]	Triterpenoids, polysaccharides	Immunomodulation, antioxidation, anti-proliferative, pro-apoptotic, and anti-angiogenic functions	Edible [93]
*M. lobayensis* [62]	PSPC	Antitumour and immunomodulatory effect	Edible [94]
*P. ferulae* [53]	PFPS	Immunomodulation, tumour growth inhibition	Edible [93]
*F. velutipes* [69]	FIP-fve	Induces CD8+ T-cell immune responses	Edible [93]
*C. sinensis* [95]	Cordycepin, polysaccharides, sterols	Antitumour and immunomodulatory effect	Edible [96]
*P. ostreatus* [88]	Ergosterol peroxide	Radiosensitizer	Edible [93]
*L. squarrosulus* [89]	Purified peptide	Increases cisplatin-induced cytotoxicity	Edible [97]

PSK: polysaccharide-K; PSP: polysaccharopeptide; ROS: reactive oxygen species; PSPC: polysaccharide-protein complex; PFPS: *Pleurotus ferulae* polysaccharides; FIP-fve: fungal immunomodulatory protein.

## Data Availability

The data presented in this study are available on request from the corresponding author.

## Supplementary Materials

The following supporting information can be downloaded at: https://www.mdpi.com/article/10.3390/life13010244/s1, References [95,101,103,105,106,112] are cited in the supplementary materials. Figure S1: Polysaccharide portions of the polysaccharide peptide (PSP) of COV-1 strain of Coriolus versicolor [101], Figure S2: Structure of major saccharide portion of PSK, G: B-D-Glucopyranose [103], Figure S3: Structures of triterpenoids isolated from G. lucidum [105], Figure S4: Structure of lentinan [106], Figure S5: Structure of cordycepin [95], Figure S6: Structuce of *L. squarrosulus*—octahydropyrrolo[1,2-a]pyrazine [112], Figure S7: Structuce of *L. squarrosulus*—fumaric acid, monochlorid, 6-ethyloct-3-yl ester [112].
