# Supplementary material for: Exploring the Bioactive Mycocompounds (Fungal Compounds) of Selected Medicinal Mushrooms and Their Potentials against HPV Infection and Associated Cancer in Humans
Source: Life (Basel). 2023 Jan 16;13(1):244. doi: 10.3390/life13010244 (PMC9861011; doi:10.3390/life13010244)
Supplement: Supplementary file 1 [file life-13-00244-s001.zip › life-2048413-supplementary.pdf]

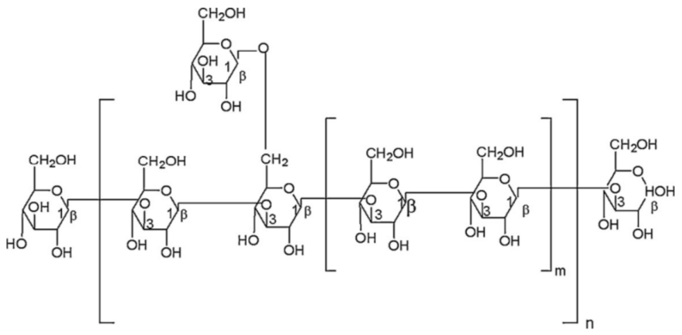

Figure S1 Polysaccharide portions of the polysaccharide peptide (PSP) of COV-1 strain of Coriolus versicolor [101].

PSK

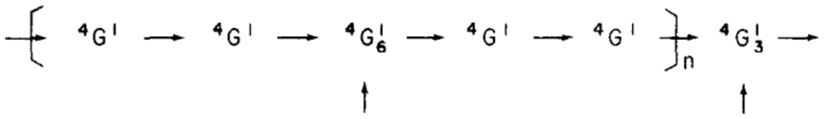

Figure S2 Structure of major saccharide portion of PSK, G : B-D-Glucopyranose [103].

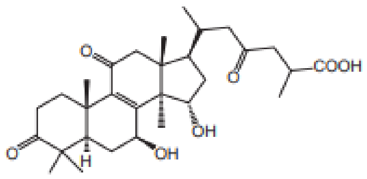

Ganoderic acid A

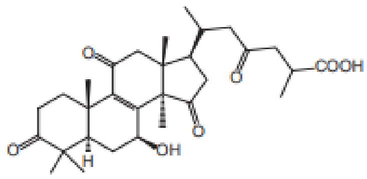

Ganoderic acid D

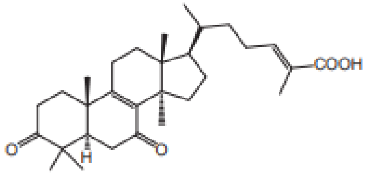

Ganoderic acid DM

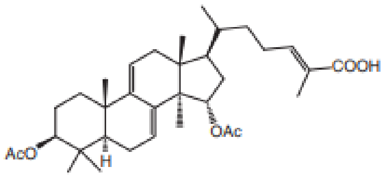

Ganoderic acid Me

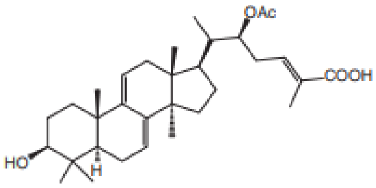

Ganoderic acid S

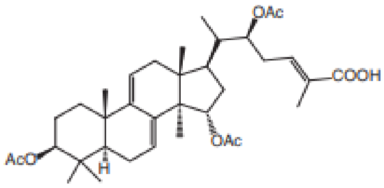

Ganoderic acid T

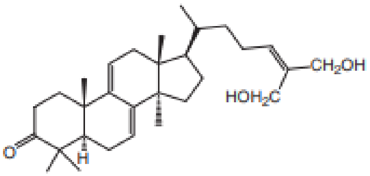

Ganoderiol F

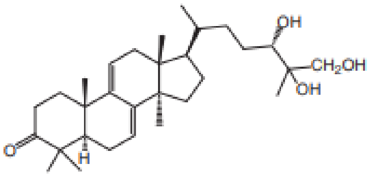

Ganodermanotriol

Figure S3 Structures of triterpenoids isolated from G. lucidum [105].

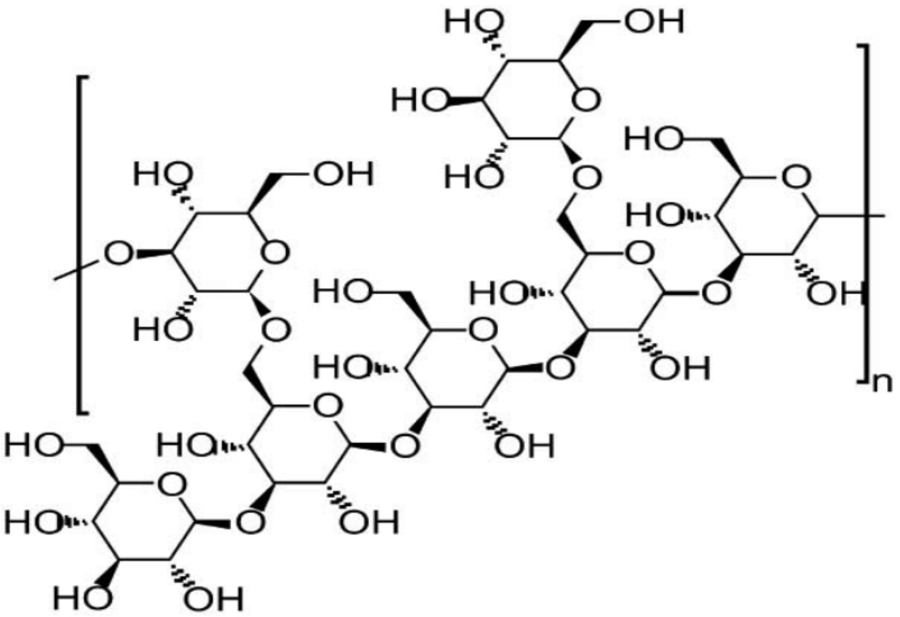

Figure S4 Structure of lentinan [106].

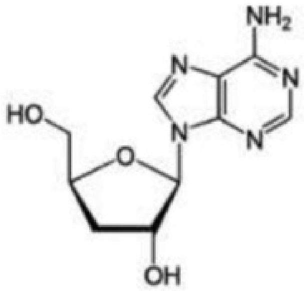

Figure S5 Structure of cordycepin [95].

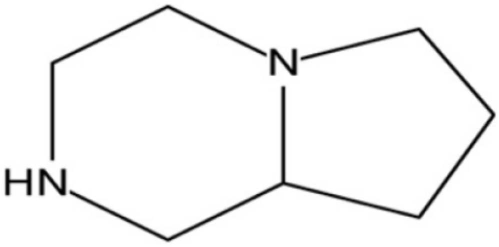

Figure S6 Structuce of L. squarrosulus - octahydropyrrolo[1,2-a]pyrazine [112].

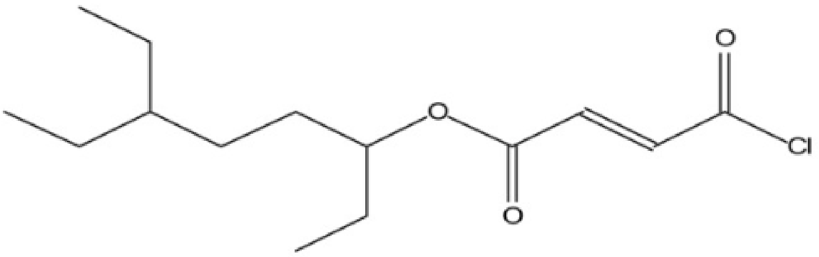

Figure S7 Structuce of L. squarrosulus - fumaric acid, monochlorid, 6-ethyloct-3-yl ester[112].
